# 3D-Bioprinted Oil-Based Hydrogels: A Sustainable Approach for Bone and Dental Regeneration

**DOI:** 10.3390/ijms26083510

**Published:** 2025-04-09

**Authors:** Syafira Masri, Nurulhuda Mohd, Noor Hayaty Abu Kasim, Masfueh Razali

**Affiliations:** 1 Department of Restorative Dentistry, Faculty of Dentistry, Universiti Kebangsaan Malaysia, Kuala Lumpur 50300, Malaysia; syafiramasri@ukm.edu.my (S.M.); nurulhuda.mohd@ukm.edu.my (N.M.); 2 Department of Restorative Dentistry, Faculty of Dentistry, University of Malaya, Kuala Lumpur 50603, Malaysia; nhayaty@um.edu.my; 3 Mesomorph Worldwide Sdn. Bhd., Kuala Lumpur 52200, Malaysia

**Keywords:** hydrogels, dental, bone, tissue engineering, 3D bioprinting, oil-based hydrogel, natural oil, biomaterials

## Abstract

Recent advancements in 3D bioprinting technology have transformed the development of complex tissue scaffolds, offering significant potential for applications in bone and dental regenerative medicine. Oil-based hydrogels have garnered considerable interest owing to their tunable mechanical properties, biocompatibility, and ability to facilitate cell adhesion, proliferation, and differentiation. This review provides an in-depth review of recent research regarding the utilization of oil-based hydrogels in bone and dental tissue development, focusing on the 3D bioprinting strategies. The review investigates the biological efficacy of the diverse oils used in hydrogel formulations, as well as their physicochemical properties, in promoting osteogenesis and dental tissue regeneration. Significant results from both in vitro and in vivo research are examined, emphasizing their capacity to sustain biological functions and promote tissue regeneration. Challenges such as hydrogel stability, printability, and cytotoxicity efficiency are thoroughly examined, along with strategies to improve these materials for translational and clinical applications. This study highlights the revolutionary potential of oil-based hydrogels in enhancing bone and dental regenerative medicine, providing insights into their current status, as well as future research and development pathways.

## 1. Introduction

The prevalence of abnormalities in oral and maxillofacial tissues is increasing due to factors such as maxillofacial trauma, malignancies, periodontal disease, and an aging population. Restoring oral and maxillofacial functions and improving aesthetics are of the utmost importance for patients who have experienced tooth loss, congenital abnormalities, traumatic deformities, or other dental conditions as stated earlier. Hence, tissue restoration holds significant value in dental medicine, including oral and maxillofacial surgery, periodontics, orthodontics, endodontics, and routine clinical practice.

In dentistry and other medical fields, conventional treatments for these abnormalities such as autografts, allografts, and synthetic graft materials are commonly used. However, these treatments carry inherent risks, including the potential for infection and graft rejection [1]. Additionally, these approaches have limitations such as the risk of secondary trauma, inflammatory reaction, and poor biocompatibility [2]. To address these challenges, tissue engineering emerged in the 1990s as a promising solution by combining cell biology and material science to regenerate tissues and organs in laboratory settings and within living organisms.

Tissue engineering and regenerative medicine in dentistry hold significant promise for restoring and regenerating dental tissues, leading to a revolutionary change in traditional dental therapies [3,4,5]. The process of regenerating dental tissues is particularly challenging due to the intricate hierarchical structures, functional demands, and inherent limits in their regenerative capacity. Progress in biomaterials, cell-based therapies, and tissue engineering strategies have offered innovative solutions to these difficulties [6,7]. The field of dental tissue engineering aims to mimic the inherent regeneration mechanisms that take place during tooth formation and healing. The process entails utilizing biocompatible scaffolds, growth factors, and stem cells to provide an ideal microenvironment that facilitates cell adhesion, proliferation, and differentiation [8,9]. Scaffolds serve as three-dimensional (3D) structures that offer structural support and direct the development and arrangement of cultured cells. These scaffolds promote the development of functional tissue constructs by imitating the composition of the extracellular matrix (ECM) and the architecture of tooth tissues.

The development of 3D bioprinting in recent years has significantly transformed the fabrication of intricate structures for dental tissue engineering. This additive manufacturing approach enables precise customization of scaffold architecture, porosity, and spatial arrangement of cells and bioactive substances [10]. 3D bioprinting utilizes computer-aided design (CAD) software (version 2023 (Autodesk Inc., San Francisco, CA, USA)) and a sequential deposition of biomaterials to create patient-specific scaffolds for patients. These scaffolds exhibit unique mechanical and biological characteristics. Additionally, 3D bioprinting technology also allows rapid prototyping and delivering of cell-laden scaffolds at high precision and resolution. The tissue can also be fabricated with a highly controllable microenvironment.

### 1.1. Pathophysiology of Dental Tissue and Periodontitis

The pathophysiology of human teeth refers to the functional alterations that arise in teeth as a result of illness or damage. Consequently, different tooth groups (incisors, canines, premolars, and molars) exhibit distinct roles and possess varying tooth structures [11]. The three primary dental tissues are enamel, dentin, and cementum. The enamel, or the crown, covers the visible section of the tooth [12]. The main components of the enamel are hydroxyapatite crystals, which give it its hardness and strength. It lacks living cells and is incapable of regeneration once it has been produced. The primary roles of enamel are to protect the underlying dentin and provide a smooth surface for chewing and articulation. In addition, enamel is known as the most densely mineralized and hardest tissue in the human body, capable of withstanding stretching, compressing, and loading [11].

The majority of the tooth is made up of dentin, which is a living tissue consisting of collagen and minerals. Dentin has a chemical makeup comparable to bone, but it is tougher due to a higher mineral content [12]. Dentin, unlike enamel, is a vital tissue that, along with pulp, constitutes the dentin–pulp complex. Dentin is located under the enamel and cementum layers of the tooth. It forms the main part of the tooth structure and is a mineralized tissue composed of hydroxyapatite crystals, collagen fibers, and tubules filled with fluid [11]. This cohesive functional module regulates tooth homeostasis and allows the teeth to respond to external stimuli [13,14].

The root surface is covered with cementum, which, together with periodontal ligaments, ensures the secure attachment of teeth to the surrounding alveolar bone. Cementum is a calcified substance that forms a protective layer around the roots of teeth. Cementum is produced by specialized cells known as cementoblasts, which originate from the dental follicle. Anatomically, the cervical area has the thinnest layer, measuring between 20 and 50 μm. As we move towards the root apex, the thickness of the layer gradually increases to a range of 150 to 200 μm.

The structural integrity of dental tissues is essential for overall tooth function. Any disruptions in this balance may result in pathological conditions. Periodontitis is a chronic, multifactorial, inflammatory illness associated with the deposition of dental plaque (which will be referred to as dental biofilm/biofilm) and marked by gradual deterioration of the teeth-supporting apparatus, including the gingiva, periodontal ligament, cementum, and alveolar bone [15,16]. Their destruction can result in gingival recession, deep probing depths, bone loss, pathological migration, and increased tooth mobility [16,17]. If left untreated, periodontitis can ultimately lead to tooth loss. While bacterial infection is the primary trigger, the severity and progression of the disease are significantly influenced by the individual’s immune response and various risk factors, including genetics, smoking, poor oral hygiene, and systemic diseases [18]. Periodontitis constitutes a considerable global health challenge, as demonstrated by the findings from the Global Burden of Disease Study (2019), which estimated that severe periodontitis affects approximately 796 million individuals, representing 9.8% of the adult population worldwide [19].

The process starts with the deposition of plaque around the teeth, leading to the formation of microbial biofilms containing bacteria, subsequently resulting in localized gingival inflammation. Failure to address this inflammatory condition can lead to its progression into periodontitis, a more advanced and destructive form of periodontal disease [20]. At this stage, the destruction of periodontal structures is driven by harmful byproducts and enzymes produced by the periodontal bacteria Porphyromonas gingivalis and Prevotella intermedia, along with *Aggregatibacter actinomycetemcomitans* and *Tannerella forsythia*. A range of bacilli, cocci, spirochetes, amoebas, and trichomonads also contribute to this process [21,22].

**Figure 1:**
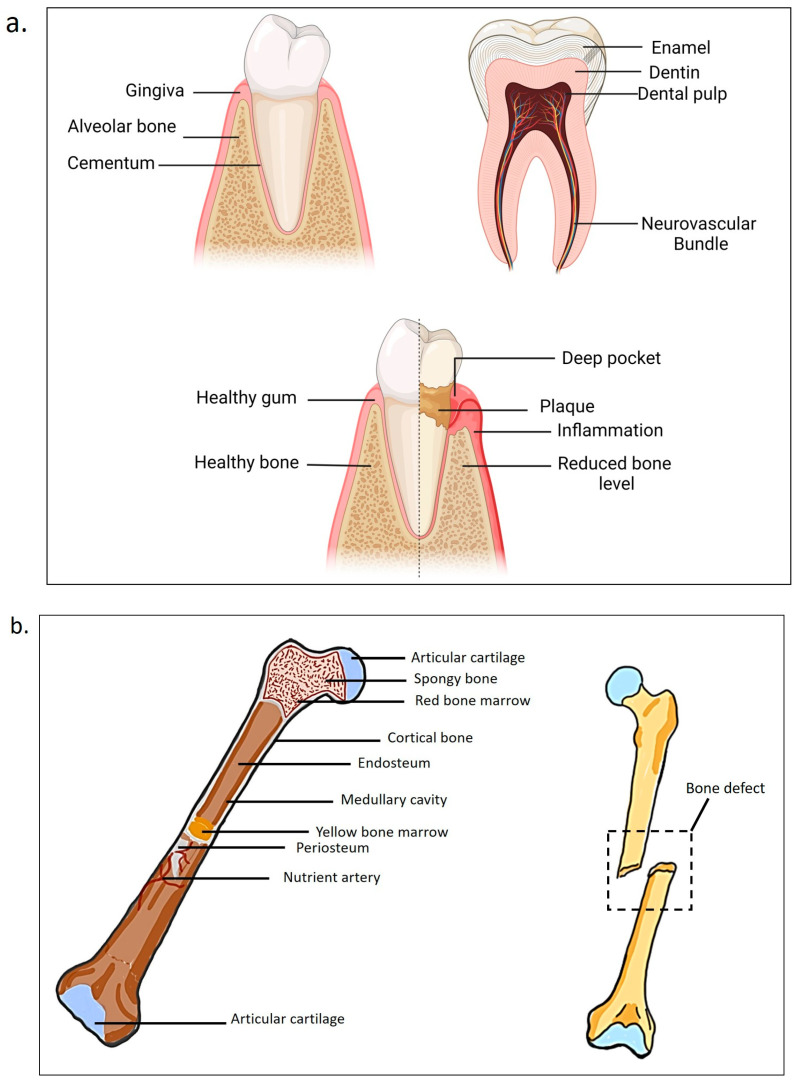
Anatomy and pathophysiology of a human tooth and bone, providing a detailed visualization of a human tooth’s anatomy and the pathophysiological changes associated with periodontitis: (**a**) the anatomy and pathophysiology of the human tooth; (**b**) the anatomy and pathophysiology of human bone. Image created using Biorender.com.

Periodontal disease progresses through four main stages [23,24,25]. The initial stage of periodontal disease, gingivitis, is characterized by the accumulation of dental plaque, halitosis, gingival inflammation, and bleeding upon brushing or flossing. It is the only reversible stage of periodontal disease and can be effectively managed through improved oral hygiene and routine professional prophylaxis. If left untreated, gingivitis may become chronic inflammation and can progress to an advanced lesion, where the inflammatory process extends into the supporting periodontal structures. Clinically, this phase presents with persistent inflammation, halitosis, and early clinical attachment loss, often manifesting as increased probing depths and radiographic evidence of mild alveolar bone loss. Although progression can be halted with appropriate intervention, the tissue destruction at this stage is irreversible. Moderate periodontitis is characterized by further clinical attachment loss, increased probing depths, gingival recession, and tooth mobility. Interproximal bone loss becomes more evident and furcation involvement may begin to appear, particularly in multi-rooted teeth. Treatment during this stage often requires comprehensive nonsurgical periodontal therapy and, in many cases, surgical intervention to achieve pocket reduction and access for debridement. In advanced periodontitis, there is severe destruction of the periodontal attachment apparatus, with 50–90% bone loss, deep periodontal pockets, suppuration, significant tooth mobility, and masticatory discomfort. Patients may present with migration of teeth and pronounced halitosis. Without timely and appropriate treatment, this stage can lead to tooth loss and may necessitate prosthetic rehabilitation. The primary goal of management at this stage is to control disease progression and maintain the remaining dentition through rigorous periodontal therapy and long-term maintenance. Figure 1 below illustrates the anatomy and pathophysiology of a human tooth, offering a comprehensive depiction of its structure and the pathophysiological alterations linked to periodontitis.

The primary objective of periodontal therapy is to restore the hierarchical structure of lost or impaired hard (alveolar bone and cementum) and soft tissues (gingiva and periodontal ligament) resulting from periodontitis. Current regenerative treatment approaches, including guided tissue regeneration, bone grafts, and bone substitutes and the application of bioactive substances, often lack consistent effectiveness in restoring the intricate hierarchical structure of the supporting tissues in many clinical scenarios [26]. Despite these advancements, the successful restoration of normal periodontal tissue architecture through current regenerative treatments remains a challenging and unpredictable clinical issue. As an alternative, tissue engineering has emerged as an effective technique for periodontal regeneration [27]. Bioprinting utilizes a bioink to encapsulate cells, which are subsequently extruded through a dispensing tip to place strands at specified locations [28]. 3D-bioprinted hydrogels provide a customizable scaffold that replicates the natural extracellular matrix, thereby supporting cell attachment, proliferation, and tissue integration, resulting in a promising solution.

### 1.2. Pathophysiology of Bone Tissue

Next, our skeletons play a crucial role in both mechanical and non-mechanical functions. The human skeleton consists of 206 bones and is categorized into the axial skeleton, which comprises the skull, hyoid, sternum, ribs, and vertebrae, and the appendicular skeleton, which includes the bones of the limbs and pelvis [29]. Mechanically, they provide a structural framework and a secure basis for human movement, producing mechanical rigidity and kinematic connection throughout the body [30]. This is accomplished by offering skeletal muscle attachment points that function as leverage and platforms for action, contraction, and force production, while also protecting the brain, spinal cord, and internal organs. Apart from that, beyond its mechanical functions, bone serves as a vital reservoir for mineral storage and plays a crucial role in regulating blood levels of calcium and phosphorus. It also supports the production of blood cells, helps defend against acidosis, and has the ability to absorb or capture potentially harmful minerals [30,31,32].

The bones consist of two components: the cortical bone, which is thick and solid, covering the marrow area, and the trabecular bone, characterized by a honeycomb-like network of trabecular plates and rods inside the bone marrow compartment [33]. Cortical bone has an external periosteal surface and an internal endosteal surface. The periosteum is a fibrous connective tissue layer that protects the outer cortical surface of the bone, except for joints where the bone is covered by articular cartilage. Trabecular osteons are commonly referred to as packets. Trabecular bone consists of plates and rods with an average thickness ranging from 50 to 400 mm [34].

Trabecular osteons exhibit a semilunar morphology, typically measuring around 35 mm in thickness, and are structured from concentric lamellae. It is estimated that healthy human adults possess 14 million trabecular osteons, encompassing a total trabecular area of approximately 7 m^2^ [34]. The periosteum is a thin layer of tissue that covers the external surface of bones, excluding joint areas, and comprises blood vessels, nerves, osteoblasts, and osteoclasts. It is connected to the bone via Sharpey’s fibers, which penetrate into the bone. The endosteum is a membrane that lines the inner surfaces of bones and the bone marrow cavity, containing blood vessels, osteoblasts, and osteoclasts.

Briefly, bone undergoes continuous growth and transformation throughout one’s lifespan via longitudinal growth, radial growth, modeling, and remodeling. Longitudinal growth takes place at the growth plates during childhood and adolescence, where cartilage is converted into new bone. Modeling involves the reshaping of bones in response to mechanical forces, facilitated by osteoblasts adding new bone and osteoclasts removing old bone. With aging, bones naturally widen due to periosteal apposition of new bone and endosteal resorption of old bone. However, bone modeling occurs less frequently than remodeling in adults [35].

In order to maintain mineral homeostasis, the skeleton remodels itself to control its own maintenance and repair. This process also offers a way for rapid access to calcium and phosphate [36]. First characterized by frost, the bone remodeling cycle is a strictly controlled process that replaces old and injured bone with new bone. This takes place in a basic multicellular unit (BMU), which is controlled by osteocytes and includes osteoclasts, osteoblasts, and capillary blood supply. The BMU functions on the surface of trabecular bone, forming and replenishing small holes known as Howship’s lacunae [37]. The BMU in cortical bone creates a cutting cone that penetrates through the bone, removing damaged areas. During this process, new bone is deposited around the tunnel walls, leaving a blood supply in the Haversian canal of the new osteon. A cell layer known as the bone-remodeling compartment (BRC) covers the BMU, ensuring that osteoclasts and osteoblasts work in close coordination throughout the process.

The complex pathophysiology of bone, characterized by a balance between formation and resorption, is essential for sustaining skeletal integrity. When this balance is disrupted due to trauma, infection, or disease, it can result in the formation of bone defects which pose considerable challenges for regeneration. Bone loss, defects, insufficient vascularization, soft-tissue damage, inadequate mechanical stability, infections, and tumors are significant challenges to successful bone healing [38]. Currently, autologous bone grafts and the transplantation of xenogenous and heterologous mineralized matrixes are used as treatments [39]. However, in certain circumstances, treatment may include the use of biomaterial implants such as polymers, metals, and ceramics. Frequently, these treatments are insufficient, and it is common for orthopedic surgeons to explore novel therapeutic alternatives capable of restoring the physiological properties of bone and cartilage tissue [40]. In tissue engineering, additive manufacturing (AM) technology, often known as 3D printing, has significant advantages that make it a viable option for bone tissue development. Bone regeneration is complicated because it requires a variety of molecular, cellular, metabolic, and mechanical stimuli. To promote bone regeneration, porous bone tissue engineering scaffolds must have the necessary form, pore size, porosity, degradability, biocompatibility, mechanical qualities, and intended cellular responses [41,42].

### 1.3. D Bioprinting for Medical and Dental Tissue Engineering

Conventional techniques for fabrication of scaffolds, such as, gas foaming, phase separation, and fiber bonding, do not provide precise control over the arrangement of cells and the appropriate ECM [43,44]. Biomedical engineering has brought forth developments that have presented 3D printing as a cutting-edge technique for rapid and precise reconstructing of injured tissues or organs. The limitations of traditional scaffolds have been solved by 3D bioprinting technology, which allows the development of 3D structures with loaded cells that resemble in vivo models [45]. In additive manufacturing, particularly 3D bioprinting, complex structures are designed using computer-aided design (CAD) software and fabricated layer by layer. When the printed materials incorporate living cells, the process is termed 3D bioprinting. Common techniques include inkjet, extrusion-based, laser-assisted bioprinting, and stereolithography.

Various systems are employed to produce bioink droplets in inkjet bioprinting, including acoustic, piezoelectric, hydrodynamic, electrostatic, magnetic and thermal processes, as well as microvalves. The printer head is coordinated with a motorized stage, and the 3D structures are constructed by scanning over each layer. This method often requires bioinks with low viscosity, ranging from 3.5 to 12 mPa/s. It is characterized by its speed and high cell viability after printing, which exceeds 85% [46]. In extrusion-based bioprinting, the bioink is subjected to mechanical or pneumatic force, which compresses it into a nozzle. This approach enables the use of bioinks across a broad spectrum of viscosities, ranging from 30 mPa/s to 60 × 10^7^ mPa/s [47]. The 3D structures are created by depositing lines or tiny beads of bioink and moving the printer head in a pattern across the platform. Subsequently, the printer head undergoes movement in the Z axis, enabling the creation of each layer in a sequential manner. The procedure is rapid, and the cell viability after printing remains at a high level of around 80% [45]. Continuous advancements in technology have led to the development of several novel printing technologies based on extrusion-based printing. These include shear-thinning printing, coagulation bath printing, support bath printing, coaxial extrusion printing, sacrificial printing, and others.

#### 1.3.1. Laser-Assisted Bioprinting (LAB)

Laser-assisted bioprinting (LAB) involves the application of a bioink with a viscosity ranging from 1 to 300 mPa/s onto a ribbon that is coated with a thin metallic coating, such as gold or titanium [48]. However, other research revealed that LAB has the capability to function with bioinks that have greater viscosities, which may range from 1 to 8000 mPas [49]. Since this printing process does not require nozzles, there are no issues with nozzle blockage. The setup comprises three main components: (i) a laser generator, (ii) a laser path adjustment system consisting of mirrors and lenses to concentrate the laser beam, and (iii) a cell transfer system consisting of a ribbon (a supportive structure with a gold or titanium layer on its bottom surface to absorb laser energy), a layer of bioink containing hydrogel or cells beneath it, and a substrate on which the ribbon prints [50]. The laser beam’s concentration on the ribbon causes the energy absorption layer to absorb energy, generating pressure on the bioink and forming a bubble. The pressure exerted on the bubble leads to the generation of an ink droplet, which is then placed into the underlying substrate. This method provides exceptional cell viability along with high precision and resolution [51].

**Figure 2:**
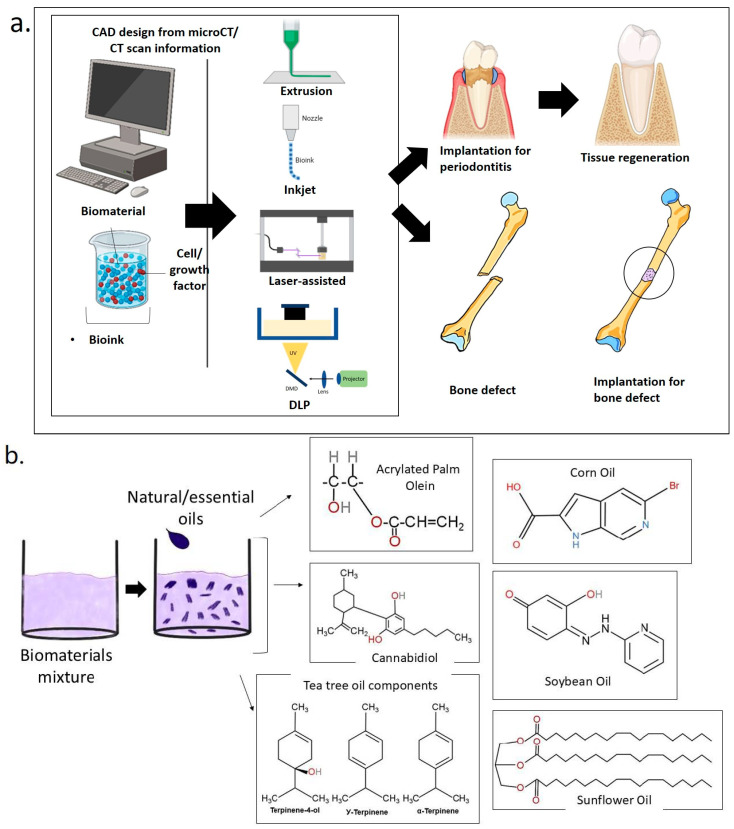
Schematic overview of 3D bioprinting techniques and their applications in periodontal and bone tissue regeneration. (**a**) The illustration shows the process of 3D bioprinting, starting with CAD design and the preparation of bioink or biomaterial inks (pink sphere represents cell/growth factors and blue sphere represents biomaterial component), and continuing through various bioprinting methods, including extrusion-based, inkjet-based, and laser-assisted techniques. The fabricated constructs are subsequently employed for implantation in the regeneration of periodontal tissue and the repair of bone defects, demonstrating their potential in the fields of dental and bone tissue engineering, and (**b**) the incorporation of natural and essential oils into biomaterial mixtures for 3D bioprinting applications. Image created using Biorender.com.

Recent studies have shown the importance of utilizing LAB for the prevascularization of bone tissue-engineered structures [52]. A specialized laser workstation was utilized to precisely deposit endothelial cells onto a ‘biopaper’, which served as the substrate. The biopaper was composed of a collagen hydrogel membrane that had been previously seeded with osteoprogenitor cells. The optimization of printing settings, cell densities, and overlay conditions was conducted to improve the fabrication of microvascular networks with a specific architecture in a laboratory setting. In this study, the researchers used in situ bioprinting to transplant endothelial cells into crucial bone calvarial lesions in mice [53]. The findings demonstrated that the bioprinting of endothelial cells enhanced the density of blood vessels in bone defects and facilitated the process of bone repair. This study also provided the initial evidence that the process of bone healing may be influenced by manipulating the arrangement of cell placement. Although the results were promising, the experimental settings did not result in complete bone regeneration. Figure 2 illustrates the process of implanting scaffolds in cases of periodontitis with 3D bioprinting technology. The process begins with the development of a computerized model of periodontal defects exhibiting characteristics of periodontitis. This model directs a 3D printer to construct a hydrogel scaffold incrementally. The hydrogel is subsequently implanted in the defects to facilitate tissue regeneration.

#### 1.3.2. Extrusion-Based 3D Bioprinting

In extrusion-based 3D bioprinting, it allows the precise control of the diameter and morphology of the scaffold, enabling the fabrication of well-defined 3D structures. Apart from that, this technique offers precise printing, rapid speed, and low cost [5]. Generally, this method will utilize mechanical force to drive the bioinks through a nozzle [54]. Extrusion-based bioprinting employs either a pneumatic or a piston/screw mechanism to dispense a continuous flow of bioink. The pneumatic method employs compressed air, while the mechanical variant utilizes a screw or piston to release the bioink from the nozzle. This approach is frequently used to develop multilayer structures in tissue engineering due to the diverse selection of biomaterials utilized in the printing process, including both natural and synthetic polymers, as well as cell-laden hydrogels and aggregates of cells [55,56].

Moreover, the dispensing pressure and shear stress produced by the system may influence cell viability [38]. The optimal viscosity of the biomaterials using the extrusion approach ranges from 30 to 60 × 10^7^ mPa/s [57]. Apart from that, one of the limitations of the extrusion technique is the challenge in attaining adequate mechanical stability and structural integrity in large free-form structures. Thus, the selection of biomaterials, according to their viscosities, is essential to avoid nozzle clogging during extrusion. The primary challenge in 3D bioprinting is the restricted availability of appropriate inks.

#### 1.3.3. Inkjet-Based 3D Bioprinting

Inkjet bioprinting, or the drop-on-demand (DOD) approach, employs heating reservoirs, piezoelectric actuators, and electrostatic or electrohydrodynamic processes to deposit cells and/or biomaterials as droplets onto surfaces [9]. Briefly, ejection is produced by acoustic, thermal, or electromagnetic forces. Inkjet bioprinting enables the accurate placement of cells, with certain studies demonstrating the capability to print as few as one cell per droplet [58]. Thermal inkjet printing employs a heated element to initiate bubble nucleation. The bubble generates pressure accumulation within the printhead, resulting in the ejection of a droplet. The thermal element can reach temperatures ranging from 100 °C to 300 °C [59]. At first, there were concerns that elevated temperatures might harm the cells. However, studies have indicated that these high temperatures are restricted to specific areas and occur only for a short time [60,61].

In inkjet-based 3D bioprinting, the droplets will traverse the charging plates and acquire a charge. The charged droplets traverse the deflection plates, which regulate droplet positioning by modifying the electric field strength [62]. Apart from that, DOD refers to a printing technology that allows for the precise application of ink or other materials only when needed, thereby minimizing waste and optimizing efficiency. Inkjet bioprinting generates droplets only upon activation, thereby enhancing precision and optimizing ink consumption. The structure is simpler than that of continuous inkjet printing, as it does not necessitate droplet charging or ink circulation [62]. These printers typically feature multiple print heads equipped with small nozzles, some measuring as small as 18 μm [63]. Ink releases itself through regulated pressure pulses, which reduces leakage in the absence of a pulse.

In thermal inkjet printing, a heat actuator rapidly heats the ink for several microseconds, resulting in the formation of small vapor bubbles. The rapid expansion of these bubbles generates a force that expels the ink through the nozzle [62]. A cavity neck structure is incorporated to enhance this force, directing the bubble expansion toward the nozzle and inhibiting backward movement. The heating temperature typically reaches 250–350 °C to achieve a sufficiently intense bubble expansion [61]. The nozzle diameter of a thermal inkjet printer typically measures approximately 50 μm, while the diameter of the resulting droplets ranges from 30 to 80 μm [64].

Each bioprinting method necessitates bioinks possessing specific rheological properties. Inkjet printing demonstrates potential for the fabrication of 3D structures that replicate human tissues through the patterning of hydrogels and living cells. Controlling the deposition of multiple bioinks presents challenges due to variations in droplet ejection, resulting in less complex artificial tissues. In inkjet-based bioprinting, the bioinks used exhibit low viscosity (<10 mPa·s) and reduced cell densities (<16 × 10^6^ cells/mL). This method yields cell viabilities approximately at 90% [65].

## 2. A Sustainable Approach for Bone and Dental Regeneration

Natural plant and essential oils have attracted significant interest in regenerative medicine owing to their bioactive features, biocompatibility, and sustainability. Natural compounds recognized for their anti-inflammatory, antioxidant, anticancer, and antibacterial properties have been explored for their potential bone- and dental-healing abilities [66]. Additionally, the utilization of vegetable oil as a renewable resource in the synthesis of polymeric biomaterials offers numerous benefits. Plant oils are renewable, environmentally friendly, biocompatible, and relatively inexpensive. In human dentistry, plant extracts and essential oils have historically proven effective as agents for combating plaque and cleaning teeth [67]. These phytochemicals offer a viable approach for the prevention and treatment of dental caries, oral inflammation, and other oral infections, and may serve as a powerful alternative to antibiotics.

Essential oils are intricate combinations of many molecules, predominantly generated from aromatic and medicinal plants, including terpenoids, aromatic constituents originating from phenol, and aliphatic components. Some essential oils possess antimicrobial and antioxidant properties, and when combined with other substances, they can exhibit bacteriostatic, bactericidal, and anti-inflammatory effects [68]. Consequently, their application in bone and dental regeneration increases, particularly when they can be easily incorporated into hydrogel formulations. This development creates new opportunities for the delivery of active compounds at the mucosal membrane level, especially for antibacterial, antioxidant, and anti-inflammatory agents, all of which are witnessing significant growth.

### 2.1. Soybean Oil

Soybean oil (SBO) has gained widespread popularity among numerous vegetable oils due to its large number of carbon–carbon double bonds, which produce a variety of monomers. In the 2020–2021 crop year, soybean oil was the most produced oilseed globally, with around 362 million metric tons produced [69]. SBO is derived from the seeds of *Glycine max* and is known as one of the cost-effective primary sources of edible oil globally. The composition includes linoleic acid, oleic acid, palmitic acid, alpha-linolenic acid, and stearic acid residues, with 85% of the oil including unsaturated fatty acids [70]. The minor constituents of SBO include phospholipids (lecithin), phytosterols, and tocopherols, which are regarded as natural antioxidants that preserve the integrity of cell membranes and preserve them against harmful reactive oxygen species (ROS). However, through recent developments in genetic manipulation, a soybean seed having 83% oleic acid (rather than carrying unsaturated linolenic acid as the primary component) has been generated [71]. The oils produced from this novel seed exhibit viscosity and oxidative stability that are almost 30 times greater than those of traditional oil. Moreover, antioxidants still remain one of the most efficient and cost-effective approaches to regulating the development of oxidative rancidity and to increase the oxidative stability of oils.

In dentistry, soybean oil is epoxidized to generate epoxidized soybean oil (ESBO), used as a plasticizer and stabilizing agent. In the study by Miao et al. (2016), soybean oil (SBO)-epoxidized acrylate was identified as a novel liquid resin for the fabrication of biocompatible scaffolds using multi-dimensional stereolithography [72]. The findings demonstrated that, compared to polylactic acid (PLA) and polycaprolactone (PCL), SBO-epoxidized acrylate exhibits superior biocompatibility and improved manufacturability [72]. The SBO-epoxidized acrylate is a liquid at room temperature and does not require any reactive diluents or heating for stereolithography. The printed scaffold in this study exhibits a substantially higher level of human mesenchymal stem cell (hMSC) attachment and proliferation than poly(ethylene glycol) diacrylate (PEGDA), and it is comparable to PLA and PCL.

### 2.2. Corn Oil

Corn oil, also known as maize oil, is derived from the germ of corn. Briefly, corn oil is mostly utilized as cooking oil and also as a source for biodiesel. Additional uses of maize oil include rustproofing for metal surfaces, inks, paints, textiles, nitroglycerin, and pesticides. Additionally, it serves as a carrier for medicinal compounds in pharmaceutical applications [73]. Generally, corn has more oil content than other commercial cereals due to its larger germ. Corn is not considered as an oilseed since it contains between 3% and 5% lipids in its kernel. The germ constitutes around 9% to 11% of the kernel’s weight and contains around 80% of the lipids present in the whole kernel [74]. A total of 13 saturated, 4 monounsaturated, and 6 polyunsaturated fatty acids have been identified in corn seeds. The primary fatty acids were saturated (palmitic [C16:0] and stearic acids [C18:0]) and unsaturated (oleic [C18:1] and linoleic acid [C18:2]) [75].

Although there is increasing interest in natural oils for bone and dental regeneration, research on corn oil is still limited. Corn oil, with its abundant antioxidants and anti-inflammatory compounds, may enhance tissue repair and modulate oxidative stress in regenerative applications. Future studies should investigate its impact on cellular responses, biomaterial integration, and overall tissue regeneration to enhance understanding of its role in this field.

### 2.3. Sunflower Oil

Sunflower is one of the four primary oil commodities in the world [76]. In 2018, around 52 million tons of sunflower were farmed across 27 million hectares, with nearly all of them being utilized for oil extraction, constituting 9.0% of the global edible oil production [77]. Briefly, the non-volatile sunflower oil is derived from the seeds of *Helianthus annuus* and it is commonly recognized as a “pioneer crop for saline lands”. Sunflower oil was often utilized as a cooking oil and an emollient in cosmetic compositions. Moreover, sunflower oil includes a substantial quantity of bioactive compounds, including vitamin E, sterols, squalene, and other aliphatic hydrocarbons, which are attributed to its biological effects [78]. Native Indians in the American continent have utilized sunflower as an ethnomedicinal plant for centuries. Among other things, the seeds and other components of the plants may be employed to treat cardiac diseases, malaria, viral infections, bronchial and pulmonary infections, coughs, and whooping cough [79].

Allelopathic chemicals, antioxidants, anti-inflammatory, anti-hypersensitive, antibacterial, and anti-hyperglycemic components were found in the seeds, receptacles, roots, and foliage leaves of this crop, making it a significant source for ethnomedicine around the world. Sunflower oil mostly consists of fatty acids, with stearic acid as the predominant component, along with a significant number of unsaturated fatty acids, mainly oleic and linolenic acids. Furthermore, extracts from sunflowers comprise complex components including diterpenes, carboxylic acids, aldehydes, steroids, polyphenols, vanillic acid, ferulic acid, trans-caffeic acid, coumaric acid, nicotinic acid, and many aromatic compounds. Generally, sunflower oil is a significant source of tocopherols and sterols with powerful antioxidant properties, while its entire seeds include micronutrients such as zinc, selenium, and iron that may enhance human immunity [80,81].

In tissue engineering, derivatives of sunflower oil can be integrated into hydrogel scaffolds that mimic the ECM of bone. These scaffolds facilitate an optimal environment for osteoblast proliferation and development. Sunflower oil-based hydrogels can be loaded with bioactive compounds like hydroxyapatite or growth factors to help with osteogenesis and periodontal regeneration [82]. Their porous and hydrophilic nature promotes nutritional and cell activity, hence aiding bone, dental, and periodontal regeneration. Composite biomaterials derived from sunflower oil have been synthesized via thiol–ene Michael reaction and reinforced with varying concentrations of cellulose nanocrystals, exhibiting promising applications as biosensors and scaffolds for supporting cell proliferation [82].

### 2.4. Tea Tree Oil

*Melaleuca alternifolia* essential oil, often known as tea tree oil (TTO), is extensively utilized topically in Australia owing to its many therapeutic effects [83]. TTO has antioxidant characteristics and demonstrates extensive antibacterial activity against numerous skin and mucosal diseases, including bacteria, viruses, fungi, and protozoa [84]. The antiseptic properties of TTO are well-known, and this study demonstrated that TTO alone is particularly effective in inhibiting the growth of methicillin-resistant *Staphylococcus aureus* [85]. Nonetheless, numerous issues are associated with TTO formulations, including the oxidation of oil components, potential incompatibility and adsorption with certain packaging materials, and reduced stability when integrated into conventional emulsions, leading to phase separation, coalescence, or flocculation.

Furthermore, TTO is a complex mixture of various volatile compounds, including terpinene-4-ol (≥30%), γ-terpinene (approximately 20%), α-terpinene (approximately 8%), p-cymene (approximately 8%), α-pinene (approximately 3%), terpinolene (approximately 3%), and 1,8-cineol (≤15%). These compounds have exhibited a significant biological activity [86]. The highest concentration of 1,8-cineole is 15%, whereas the minimum concentration of terpinen-4-ol is 30%. Terpinen-4-ol is the primary active component of the complex mixture of compounds in TTO. It can be characterized by the presence of an alcoholic –OH group in its structure, followed by 1,8-cineole, which exhibits irritating properties [87].

In the field of tissue engineering, TTO has been extensively utilized in conjunction with other materials to produce hydrogels. The incorporation of TTO into chitosan and polyvinyl alcohol (CS-PVA) matrices has been shown to enhance the mechanical strength of the resulting films, as evidenced by a notable reduction in Young’s modulus and improved scaffold stability [88]. However, careful control of TTO concentration is necessary, as excessive amounts may induce cytotoxic effects. In a subsequent study, the addition of TTO to polycaprolactone/polylactic acid (PCL/PLA) composites was found to improve both biocompatibility and biodegradability, leading to faster resorption and the absence of an aggressive inflammatory response [89].

### 2.5. Cannabis Sativa Oil

*Cannabis sativa* L. is among the oldest plants traditionally used by humans for medical purposes. Despite the restricted usage of *Cannabis sativa* (CS), the medicinal sector surrounding this annual plant has expanded significantly in recent years. The utilization of cannabis as a therapeutic agent for certain disorders or alleviation of symptoms from various therapies has grown globally due to its diverse pharmacological effects and advantages for patients [90]. Briefly, CS (hemp) is a herbaceous dioecious species belonging to the Cannabinaceae family. The appealing pharmacological characteristics derive from the bioactive phytocannabinoid substances inherently found in CS. The primary constituents Δ9-tetrahydrocannabinol (THC) and cannabidiol (CBD) have distinct interactions with the human endocannabinoid system, demonstrating psychotropic and non-psychotropic effects, respectively [91]. Additionally, CO contains cannabinoids including cannabidiol (CBD) and tetrahydrocannabinol (THC), as well as terpenes and flavonoids. However, CBD is known as non-psychoactive and have anti-inflammatory, antioxidant, and analgesic qualities, whereas THC is psychoactive and has the ability to alter pain and immunological response.

In tissue engineering, cannabis oil can be added into biomaterials such as hydrogels, scaffolds, and coatings to boost their bioactivity. In addition, there is evidence indicating that cannabis can modulate molecular and cellular mechanisms associated with wound healing, possess anti-inflammatory features that diminish fibrosis and proliferate hyperproliferative keratinocytes, and expedite injury closure [92]. Furthermore, a study has proven the remarkable antibacterial efficacy of CS, mostly against gram-positive bacteria [93]. Antezana et al. (2022) demonstrated that incorporating *Cannabis sativa* oil into the GEL–ALG scaffold improved its structural stability and antioxidant activity, while showing no cytotoxic effects on 3T3 fibroblast cells [94].

### 2.6. Acrylated Palm Olein

Palm oil is one of the most appealing renewable alternatives, and its inherent biodegradability is also a desirable feature in the context of rising environmental concerns compared to petroleum-based products in terms of minimizing production costs and maximizing inputs and outputs, such as food supply, water management, energy and product recovery, and waste treatment. In Malaysia, palm oil and jatropha oil have been generated from relatively affordable and renewable resources for biodiesel manufacturing. Nationwide, the third-largest plantation crop is *Jatropha curcas* L. [95]. Briefly, palm oil is produced as a liquid from the orange-red fleshy mesocarp of oil palm fruits, which are generally made of 45 to 55% oil [96]. Palm oil comprises a complex blend of triglyceride molecules and it is constituted of fatty acids, including palmitic, stearic, myristic, oleic, and linoleic acids [97]. Palm oil comprises 50% saturated, 40% monosaturated, and 10% polyunsaturated fatty acids (PUFAs) along with a great supply of phytonutrients. Tocotrienols, bioactive components of palm oil, are useful in treating atherosclerosis and some cancers. They were also shown to be effective against oxidative damage to lipids in vitro and in vivo, as well as in neuroprotection [98].

In addition, acrylated palm olein (APO) is derived from acrylic-ester palm olein, such as epoxidized polyol (EPOo) [99]. The synthesis of acrylated palm olein (APO) was performed by ring-opening oxirane groups of the epoxidized palm olein (EPOo) using acrylic acid, resulting in APO with a high yield of 86% and molecular weight of 1750 Da [100]. APO possesses hydroxy groups that are effective for surface modification to boost the medication transport performance to the targeted organ [101]. In the recent past, palm oil has gained significant interest in tissue engineering field due to the presence of low molecular weight, ester functional groups, and amorphous nature, APO is considered to be more suitable for biomaterial applications such as nanoparticles, polymeric micelles, microspheres, and liposomes [99]. A novel APO-based photopolymer resin, manufactured using micro-stereolithography, enables the fabrication of bioactive, porous, and mechanically resistant tissue scaffolds for applications like maxillofacial prostheses and 3D elastomeric dentures.

Studies on polyhydroxyalkanoates (PHAs) generated from palm oil waste indicate its promise as a sustainable biomaterial; however, radiation methods remain underexplored in this context. In a study conducted by Rao et al. (2010), poly(3-hydroxybutyrate-co-4-hydroxybutyrate) [P(3HB-co-4HB)] was biosynthesized using cupriavidus necator cultured on palm oil waste as the carbon source [102]. Their findings demonstrated that affordable wasted palm oil is a useful source of carbon in the production of polyhydroxyalkanoates (PHAs), effectively employing cupriavidus necator via biosynthesis method [102]. This biopolymer eventually can become a new absorbable biomaterial for medicinal applications.

## 3. Hydrogels Enriched with Natural/Essential Oil for Bone and Dental Regeneration

The success of regenerative medicine is contingent upon the specific attributes of scaffolds, including biocompatibility, mechanical strength, and porosity. The research studied in this review emphasize the adaptability and promise of 3D bioprinting methods for constructing oil-based hydrogels as part of the scaffold used in bone and dental therapies. The research exhibited variations in terms of study design, types of 3D bioprinting processes employed, bioink components used, and particular outcomes pertaining to bone and dental applications. This section aims to highlight various approaches and outcomes in the use of oil-based hydrogel formulations for 3D bioprinting in bone and dental tissue engineering applications.

**Figure 3:**
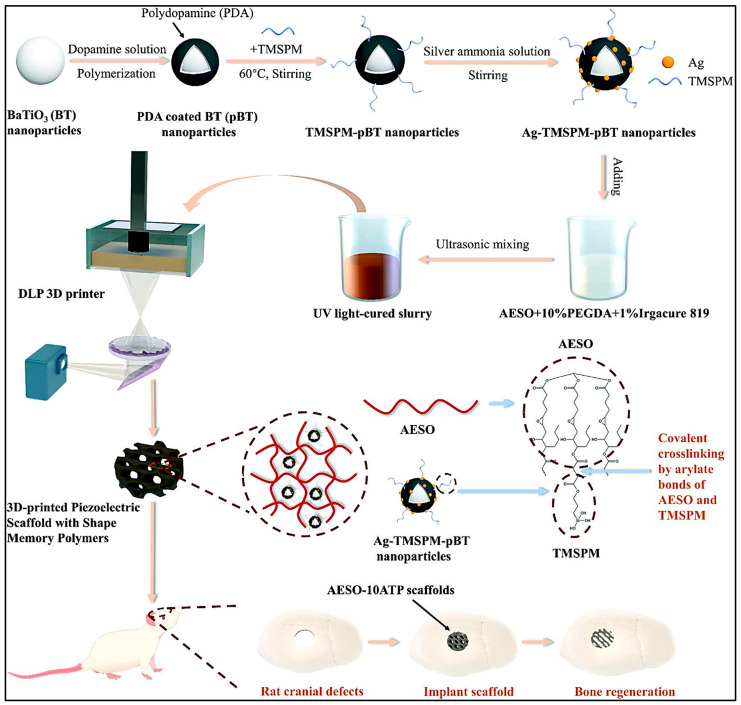
Schematic illustration of the fabrication process of 3D-printed piezoelectric scaffolds with shape memory polymers using Ag-TMSPM-pBT nanoparticles and AESO-based UV light-cured slurry for bone regeneration in rat cranial defect models [103]. Reproduced with permission from Li et al. (2023) [103]. Copyright © 2023 by John Wiley & Sons A/S. Published by John Wiley & Sons Inc.

Li et al. (2023) highlighted the utilization of AESO scaffolds embedded with piezoelectric nanoparticles in 3D printing for the purpose of bone regeneration as illustrated in Figure 3 [103]. In this study, the scaffolds demonstrated excellent biocompatibility and stimulated bone growth in both in vitro and in vivo. The study demonstrated that piezoelectric scaffolds enhanced the osteogenic differentiation of bone marrow stem cells (BMSCs) in vitro by promoting cell proliferation, alkaline phosphatase (ALP) activity, and mineral deposition. Additionally, the scaffolds facilitated new bone formation by promoting tissue integration and improving bone regeneration at the defect site in vivo. The findings demonstrate the scaffolds’ capacity to facilitate an optimal environment for bone tissue growth and repair. Moreover, the AESO-10ATP scaffolds facilitate bone regeneration via various mechanisms. Piezoelectric properties, similar to those of natural bone, produce electrical signals when subjected to mechanical stress, thereby promoting cellular activity and stimulating bone growth. Furthermore, these scaffolds facilitate the differentiation of bone marrow stem cells (BMSCs) into osteogenic cells, promoting new bone formation. The shape memory function enables scaffolds to rapidly revert to their original shape when exposed to near-infrared (NIR) light, thereby preserving structural integrity throughout the repair process. The presence of Ag-TMSPM-pBT nanoparticles enhances conductivity and promotes an environment conducive to cell attachment and proliferation, thereby promoting bone tissue regeneration. The combined effects render the AESO-10ATP scaffolds promising for the repair and regeneration of bone defects.

Expanding on the application of plant-derived oils, biomimetic porous, bioceramic scaffolds composed of hydroxyapatite and sunflower oil were developed for bone tissue engineering, demonstrating support for cell proliferation and potential for further use in bone marrow regeneration [104]. In a related effort, a novel bioink consisting of skin gelatin, zinc oxide, and clove oil was formulated specifically for extrusion-based 3D bioprinting, exhibiting strong antibacterial activity and showing considerable promise for the production of personalized biomedical constructs [105]. The study of nanocomposite scaffolds for bone tissue engineering has also been carried out with acrylated epoxidized soybean oil (AESO), in which the addition of hydroxyethyl acrylate greatly enhanced mechanical strength, printability, and osteogenic differentiation capability [106]. To support bone regeneration, Kim et al. (2022) developed hybrid cell constructs incorporating porcine bone-derived ECM, which significantly enhanced both angiogenic and osteogenic activities—highlighting their potential in promoting bone formation [107]. In a related study, Kim and Kim (2022) formulated a cell-laden bioink aimed at bone and cartilage regeneration [108]. This bioink was specifically designed to enhance cell proliferation and osteogenic differentiation within human adipose-derived stem cell constructs. A summary of various oil-based hydrogel formulations utilized across different 3D bioprinting techniques is provided in Table 1.

### 3.1. Rheological Properties of 3D-Bioprinted Oil-Based Hydrogels

The emergence of 3D bioprinting has transformed tissue engineering and regenerative medicine by facilitating the accurate construction of intricate tissue architectures. Hydrogels have emerged as a flexible and promising material in 3D bioprinting due to their biocompatibility, high water content, and capacity to replicate the ECM. Recent breakthroughs have integrated oil-based hydrogels into this field, presenting distinctive features and benefits compared to conventional aqueous hydrogels. Oil-based hydrogels, distinguished by the integration of oil phases within a hydrogel matrix, have unique rheological features that markedly affect their printability and efficacy. Rheology, the study of material flow and deformation, is essential in determining the extrusion, deposition, and solidification of hydrogels during bioprinting. Understanding these rheological qualities is crucial for improving the printing process, attaining the intended structural resolution, and ensuring the functional integration of the printed structures.

In a related investigation, the rheological properties of two hydroxyapatite formulations were assessed—one incorporating sunflower oil and the other containing Pluronic^®^ F-127 [104]. Both formulations exhibited shear-thinning behavior, characterized by a decrease in viscosity with increasing shear rates. This property is particularly beneficial for processes involving extrusion or flow under shear stress, as it facilitates smoother material deposition and handling during fabrication. With an increase in oil content in these formulations, there was a significant increase in viscosity, storage modulus, and yield stress. The storage modulus quantifies a material’s capacity to withstand deformation when subjected to stress, whereas the yield stress denotes the minimal level of stress needed to commence flow. The observed increases indicate that a greater amount of oil improved the strength and durability of the emulsions, making them more resistant to deformation and more resilient when subjected to stress. Nevertheless, it was noted that an excessive amount of oil caused a decrease in the concentration of HA solids in the emulsions. The decrease in solid loading restricted the potential for additional improvement in viscosity, as the concentration of the solid HA component, which contributes to the total viscosity, was lowered. Although there was a limitation, the emulsions remained stable and exhibited enhanced rheological properties overall, suggesting that the formulations remained effective for their intended uses. However, there were some restrictions on the maximum viscosity that could be achieved due to the balance between the solid content of HA.

To further improve print precision and biofunctionality, the hydrogel formulation, composed of skin gelatin (BSG), zinc oxide (ZnO), clove essential oil (CEO), and alginate, has been explored for its potential in 3D printing applications [105]. The bioink was crosslinked by utilizing a 100 mM calcium chloride (CaCl_2_) solution, which successfully initiated the process of gelation via the formation of ionic crosslinks in the alginate moiety. This process was carried out efficiently at ambient temperature, eliminating the need for additional heat or complex conditions. It demonstrated the ability to achieve a print resolution of approximately 100 µm, highlighting its suitability for high-precision bioprinting processes, thus simplifying the process of printing. The bioink had a print resolution of around 100 µm, suggesting its potential for high-resolution bioprinting applications. This high resolution indicates its capacity to be precisely extruded and deposited layer by layer, a very important consideration in building detailed and structurally sound 3D architectures. The printed structures that were obtained had complex and well-differentiated features, highlighting the bioink’s potential for fabricating complex tissue-engineered constructs.

### 3.2. Physical Properties of 3D-Bioprinted Oil-Based Hydrogels

Particularly in bone and dental tissue engineering, the physical characteristics of 3D-bioprinted oil-based hydrogels are rather important in determining whether they are suitable for biomedical uses. These qualities affect the mechanical integrity, printability, and functional performance of hydrogel scaffolds, which are crucial for preserving structural stability and facilitating cellular activity.

Therefore, Li et al. (2023) conducted a study to assess the rheological and physical characteristics of AESO and AESO-10ATP resins [103]. The incorporation of Ag-TMSPM-pBT nanoparticles into the AESO resins had a substantial impact on the properties of the scaffold. The addition of these nanoparticles resulted in a significant decrease in the water contact angle of the scaffolds from 98.8° to 62.7°. This implies a notable enhancement in the hydrophilicity of the scaffolds. The increased hydrophilicity is advantageous for biological applications where higher water affinity can promote cell adhesion and development. The scaffolds exhibited a porosity of 62.42 ± 2.05%, which closely aligns with the theoretical porosity value of 65.43%. The close-to-match observed in this study highlights the exceptional accuracy of the DLP 3D printing process. It demonstrates that the system is capable of successfully replicate intricate scaffold designs while maintaining the structural properties.

Additionally, two hydroxyapatite formulations were investigated for their suitability in fabricating 3D-printed scaffolds with macroporous architectures [104]. The study aimed to optimize scaffold structure for enhanced performance in bone tissue-engineering applications. One formulation involved the use of sunflower oil, while the other formulation utilized Pluronic^®^ F-127. Both formulations exhibited shear-thinning properties, indicating that their viscosity decreased under the influence of shear stresses. This characteristic makes them well-suited for extrusion-based 3D printing, as it allows the material to flow smoothly through the nozzle while retaining its shape during deposition. It was discovered that both the hydroxyapatite slurry and the emulsion-based inks were effectively printed into scaffolds with well-defined macroporous architectures. The hole diameters were significant, with horizontal pores ranging from 300 to 400 μm and vertical pores at roughly 100 μm between the struts. These pores are crucial for tissue engineering scaffolds since they facilitate cell infiltration, nutrition delivery, and waste removal. The formulation, characterized by a greater oil content, displayed microporous characteristics on the surface of the struts, with bigger pore sizes and areas in comparison to the F100 formulation that utilized Pluronic. The improvement in the surface structure is probably caused by the existence of oil, which may generate more empty spaces when printing or drying. The microporous strut surfaces enhance the total surface area, potentially enhancing cell adhesion and proliferation, which is crucial for successful tissue regeneration. Therefore, the study emphasizes that adjusting the composition through modifying the amount of oil can directly impact the microscopic features of the printed scaffolds.

Furthermore, a composite bioink consisting of skin gelatin (BSG), zinc oxide (ZnO), clove essential oil (CEO), and alginate was produced by Ahmed et al. (2020) [105]. In this study, calcium chloride (CaCl_2_) solution was employed for crosslinking to achieve structural stability, resulting in the formation of a solid gel from the alginate. SEM analysis demonstrated that the addition of CEO resulted in an increased number of holes in the bioink. The enhanced permeability facilitated the enhancement of the precision of the fabricated framework, leading to a more seamless and precise layer-by-layer assembly in the process of 3D printing. These characteristics enhance the suitability of the bioink for accurate and durable scaffold formation.

Similarly, bioinks composed of gelatin–alginate and *Cannabis sativa* oil have been investigated for their structural and biological potential [94]. The construct exhibits a compact and smooth surface and a highly porous structure, with fine, well-defined pores averaging 15.0 ± 2.3 μm in diameter as observed via SEM analysis. These structural features suggest potential suitability for a range of biological applications such as cell infiltration and nutrient diffusion. Rapid degradation was demonstrated within 5 h by the total disintegration of the *Cannabis sativa* oil-free gelatin–alginate scaffold. After 20 h, the scaffold that had been treated with *Cannabis sativa* oil was still only 20% lighter than when it had been initially applied. This indicates that the scaffold was able to withstand degradation better after adding *Cannabis sativa* oil, resulting in a longer lifespan than the scaffold that was not oil-treated.

Kim and Kim (2022) examined the impact of incorporating mineral oil (MO) into a bioink made of carboxymethyl alginate (CMA) on the characteristics of the scaffold [108]. The scanning electron microscopy (SEM) images showed that the CMA/MO scaffolds had a porosity of 97.9 ± 0.3%, which was greater than the porosity of scaffolds constructed solely from CMA, which was 96.4 ± 0.3%. The increased porosity of the CMA/MO scaffolds suggests that the addition of mineral oil to the bioink resulted in a more porous structure. Increased porosity is frequently advantageous in scaffolds utilized for tissue engineering since it promotes cell infiltration, nutrient exchange, and overall scaffold efficacy. The wettability tests consisted of evaluating the permeability of the scaffolds by employing a solution of fluorescently labelled dextran (FITC-conjugated) and measuring the speed at which the solution could penetrate the scaffolds. The presence of CMA/MO scaffolds facilitated a faster penetration of the dextran solution in comparison to the scaffolds made of CMA alone. The CMA/MO scaffolds demonstrated a greater protein absorption capability in comparison to the CMA-only scaffolds. Table 2 below highlights the key properties of several oil-based hydrogels utilized in 3D bioprinting for bone and dental tissue synthesis. The table highlights the types of hydrogels, crosslinking methods, rheological properties, physical characteristics, and chemical characterizations.

### 3.3. Chemical Characterization of 3D-Bioprinted Oil-Based Hydrogels

The distinctive formulation of oil-based hydrogels imparts various chemical characteristics that affect the behavior, stability, and effectiveness of bioprinted objects. Understanding these chemical features is essential for enhancing the design and utilization of oil-based hydrogels in 3D bioprinting. These features influence the material’s compatibility with biological tissues, its ability to facilitate cellular functions, and its overall stability and efficacy in diverse situations.

In addition to FTIR, X-ray diffraction (XRD) can be used to analyze hydroxyapatite (HA) films within ZnO-reinforced clove essential oil (CEO)-based hydrogels, allowing for the identification of distinctive diffraction peaks in the scaffolds [105]. These are accompanied by comparable amide bands across samples, although variations in peak positions and intensities may be observed. However, the XRD analysis of the pure gelatin film exhibited no distinct diffraction peaks. This indicates that the gelatin film does not possess a defined crystalline structure and remains amorphous, which is typical for gelatin. Prominent diffraction peaks were observed at 2θ values of 10.4°, 12.7°, 22.8°, 32.2°, 34.8°, 36.8°, and 56.5°, indicating the formation of crystalline phases within the composite films, likely due to the incorporation of ZnO and CEO. These peaks suggest enhanced crystallinity, which may influence the films’ mechanical and thermal characteristics. FTIR analysis revealed consistent amide bands (A, B, I, II, and III) across all samples, reflecting the protein structures of gelatin and their interactions with other components. Additionally, a strong band at 1035 cm^−1^ was detected, corresponding to the asymmetric stretching vibrations of O-H groups in glycerol. This peak suggests possible interactions between the film’s structure and the O-H groups, potentially associated glycerol’s plasticizing effect or its interactions with ZnO and CEO.

Moreover, FTIR analysis has been shown to be an effective method for confirming successful polymerization in photocrosslinked oil-based hydrogels. The absence of vinyl group peaks in the post-cured FTIR spectra of AESO composites indicated complete polymerization of the acrylate components, confirming effective curing of the material [106]. The FTIR spectra of the nanocomposites indicated the absence of peaks related to vinyl groups post-curing. Vinyl groups are generally found in the unpolymerized or pre-cured condition of acrylate-based substances. Their absence in the post-cure spectrum indicates that these groups were successfully polymerized during the curing process. Another thing, FTIR analysis can also be used to identify specific components within scaffold formulations. Distinctive peaks of CS at 2924 and 2850 cm^−1^ corresponding to the stretching vibrations of –CH_2_ and –CH_3_ groups, indicated the presence of chitosan in AESO–PEGDA composites [94]. These peaks were not observed in the GEL-ALG formulation, highlighting FTIR’s usefulness in distinguishing between different scaffold compositions.

### 3.4. Thermal Properties of 3D-Bioprinted Oil-Based Hydrogels

Thermal characteristics include thermal stability, heat capacity, and thermal conductivity, which are essential for understanding material performance under different temperature environments. The interplay between the oil phase and the hydrogel matrix can profoundly influence the materials’ responses to temperature variations, hence influencing their printability, structural integrity, and biological interactions. Temperature is important in 3D bioprinting because it affects gelation, post-printing cure, and material extrusion. An in-depth understanding of the thermal characteristics of oil-based hydrogels is crucial for enhancing their application in bioprinting. Thermal stability guarantees that the hydrogel preserves its structure and functioning throughout and after printing, whereas heat capacity and thermal conductivity affect the material’s performance under varying temperature settings.

Ahmed et al. (2020) conducted a study on the thermal stability of composite films formed from bovine skin gelatin (BSG), zinc oxide (ZnO), and clove essential oil (CEO) [105]. The study identified distinct degradation stages. The thermogravimetric analysis (TGA) revealed three weight loss phases: an initial 37.35% reduction between 197 and 256 °C, a secondary 31.51% loss between 267 and 367 °C, and a final 18.41% decrease between 400 and 460 °C. Moisture and low-molecular-weight compounds within the composite matrix are the primary causes of the initial weight loss stage. The gelatin component endures thermal decomposition as the temperature increases, which in turn corresponds to the second phase of degradation. The final stage is related to the potential formation of char and the decomposition of residual organic matter. The thermal stability of the bioink was considerably improved by the incorporation of ZnO, which served as a reinforcing effect and delayed the starting point of degradation.

### 3.5. Mechanical Properties of 3D-Bioprinted Oil-Based Hydrogels

The mechanical characteristics are essential for the effective use of bioprinted hydrogels, as they affect the material’s structural integrity, functionality, and performance in biological environments. Oil-based hydrogels, defined by the incorporation of oil phases within a hydrogel matrix, exhibit unique mechanical characteristics that influence their performance during and after the bioprinting process. The qualities encompass tensile strength, compressive modulus, elasticity, and toughness properties of the scaffolds. These scaffolds must possess adequate mechanical integrity to fulfil their function successfully, even if they are not required to withstand substantial loads or provide structural reinforcement.

It is interesting to note that scaffolds composed of cellulose nanocrystals (CNCs) and corn oil demonstrated superior compressive strength and Young’s modulus, exceeding those of cancellous bone, and highlighting their strong mechanical performance [104]. In hydroxyapatite (HA) films reinforced with ZnO and CEO, tensile strength decreased from 36.9 ± 2.8 MPa to 32.7 ± 2.3 MPa, while elongation at break increased from 15.05 ± 1.3% to 19.1 ± 1.8%, reflecting a trade-off between strength and flexibility [105]. Moreover, AESO composites incorporating isobornyl acrylate (IBOA) and bioactive glass particles have shown significantly higher tensile elastic moduli with values of 689.95 ± 189.45 MPa for S20 and 645.34 ± 149.78 MPa for SP20, compared to 198.72 ± 36.65 MPa for SH20, indicating improved tensile strength in the modified composites [106]. Table 3 below summarizes the thermal stability and mechanical properties for various oil-based hydrogel formulations employed in 3D printing for tissue engineering applications.

## 4. Overview of 3D Bioprinting in Bone and Dental Applications

3D bioprinting has emerged as a revolutionary approach in tissue engineering, providing precise control over the spatial organization of cells, biomaterials, and growth hormones. Innovative technologies in additive biomanufacturing enable the creation of structures that closely resemble the architecture of dental and craniofacial tissues [109]. This technique offers new solutions for complex regeneration and repair issues in bone and dental applications, facilitating the fabrication of customize scaffolds and implants that improve tissue regeneration and functional restoration. A significant use of 3D bioprinting in bone regeneration is the fabrication of customized scaffolds. These scaffolds may be constructed to conform precisely to the geometry of bone defects or absent segments, promoting optimum integration and support for new bone formation.

Bioprinting facilitates the integration of several bioactive materials, including hydroxyapatite, collagen, and calcium phosphate, which can improve osteoconductive and promote bone tissue regeneration. Studies in dental bioprinting encompass the regeneration of whole teeth or their constituent parts. Bioprinting has the ability to generate bioengineered teeth or restore damaged dental structures through the integration of stem cells, biomaterials, and growth factors. This application shows potential for preventing tooth loss and restoring dental function. Multiple parameters related to scaffold design affect printability, including strand orientation, pore size (spacing), and layer thickness [110]. The main advantage of 3D bioprinting is its ability to spatially arrange cells within solid or semi-solid biomaterials, hence enhancing tissue regeneration [111].

Consequently, 3D bioprinting facilitates the development of highly reproducible, spatially controlled structures composed of various materials, growth factors, and cells, including synthetic bone substitutes. The advancement of 3D-bioprinted bone and dental tissues is highly significant for clinical practice, as it facilitates the reconstruction of complex-shaped bone defects by converting computed tomography (CT) or micro-CT data into printable images, resulting in patient-specific implants. In tissue engineering, these methods can regulate pore size, shape, distribution, and interconnectivity in scaffold fabrication. Moreover, in conjunction with CAD and 3D medical imaging techniques like computed tomography, 3D printing facilitates the production of personalized structures (patient-specific) [112].

This review highlights various 3D bioprinting techniques applied in tissue engineering, including DLP, extrusion-based bioprinting, and light-assisted 3D printing. A range of biocompatible oil-based hydrogels and biomaterials such as acrylated epoxidized soybean oil (AESO), soybean oil (SO), cellulose nanocrystals (CNCs), and hydroxyapatite (HA) are frequently combined with sunflower oil and corn oil for scaffold fabrication. Amongst the methods discussed, extrusion-based bioprinting emerges as the most widely utilized. This technique involves the continuous deposition of material through a micro-nozzle in direct contact with the substrate, allowing precise construction of 3D structures. [113]. Generally, the print head is capable of movement along three axes: x, y, and z. This printing technique is applicable for fabricating scaffolds with specific structures from biocompatible materials and cell-embedded hydrogels. Various extrusion systems have been employed in 3D printing, including pneumatic pressure, piston, and screw-driven mechanisms. In pneumatic pressure and piston systems, the substance is typically placed into a syringe and distributed using suitable methods [113].

Furthermore, the direct delivery of anti-inflammatory, antibacterial, and antioxidant properties to the affected site can be facilitated by the incorporation of therapeutic agents such as essential oils into the hydrogel, thereby promoting tissue regeneration and expediting the healing process. A previous study has conducted a research on 3D bioprinting parameters for periodontal ligament cells (PDLCs) within GelMA hydrogels to facilitate periodontal regeneration [28]. Optimal results were obtained with 12.5% GelMA combined with 0.05% LAP, utilizing a printing pressure of 135 kPa through a 25 G needle, followed by UV crosslinking for 20 s. The conditions resulted in stable scaffolds exhibiting high cell viability, facilitating the growth and spread of PDLCs throughout the structure over a 14-day period. The results indicate that PDLC-laden GelMA scaffolds may be beneficial for periodontal tissue repair, establishing a foundation for subsequent in vivo investigations.

Previously, a novel hydrogel has been developed by integrating bioactive gold nanoparticles (GNPs) with a 3D-printed polylactic acid (PLA) framework to enhance bone tissue regeneration [114]. The hydrogel exhibited adjustable stiffness, resembling the human mandibular condyle, and facilitated the proliferation and dissemination of human adipose-derived stem cells (ADSCs). The incorporation of cyclic RGD-conjugated GNPs (RGNPs) enhanced the expression of essential markers associated with bone formation. The results indicate that this reinforced hydrogel may serve as a viable candidate for bone tissue engineering. Apart from that, a research also has developed a hybrid system comprising a 3D-printed polycaprolactone (PCL) gyroid scaffold infused with a hydrogel composed of alginate, gelatin, and nano-hydroxyapatite, aimed at facilitating the repair of long bone defects [115]. The gyroid configuration exhibited a greater capacity for hydrogel retention compared to alternative scaffold geometries. Human mesenchymal stem cells (hMSC) demonstrated effective adhesion and survival, indicating the system’s compatibility with cellular structures. Tests indicated the formation of bone-like minerals, thereby confirming its bioactivity, while the hydrogel exhibited gradual degradation over time. The findings indicate that the PCL/hydrogel system presents a viable alternative for bone tissue repair.

### 4.1. Application of Oil-Based Hydrogels for Bone and Dental

In bioprinting, hydrogels are extensively utilized as a matrix for cell encapsulation owing to their biocompatibility, biodegradability, similarity to the natural ECM, and ability to maintain a hydrated environment, hence promoting the flow of nutrients, oxygen, and waste elimination essential for cellular development [110]. The efficacy of bioprinting and printed structures is dependent on many aspects associated with the bioinks and the characteristics of the scaffold surface. Bioinks suitable for producing successful bone replacements must possess features such as biocompatibility, biomimicry, biodegradability, and mechanical integrity [116].

Bioinks offer an evaluation of the impact of geometry and spatial organization on cellular behavior and function in vitro, which may then be integrated into in vivo models for regenerative dental applications. Currently, cell printing technology has emerged as the favored option for a novel biofabrication process, overcoming the conventional method of seeding cells onto scaffolds [9]. The main bioink materials are hydrogel-based bioprinted constructs. They have acquired prominence in recent years due to their similarity to natural ECM, uniform cell distribution inside the scaffolds, capacity to support living cells, and improvement of cell survival in a hydrated three-dimensional environment [117,118].

Hydrogels exhibit a very porous structure, which may be readily manipulated by modifying the density of crosslinks within the gel network. The increased porosity facilitates the retention of oil-based pharmaceuticals inside the hydrogel matrix through hydrophobic interactions, enabling their eventual release at a regulated rate [119]. In recent years, plant-derived essential oils have attracted significant scientific attention for their antioxidant, anti-inflammatory, and antibacterial effects [120]. Numerous studies investigate the direct application of essential oils to bone tissue, their integration as bioactive chemicals in bone scaffolds, or their utilization as coatings for bone implants [66]. Oil-based hydrogels are attracting interest in dental applications owing to their biocompatibility, mechanical characteristics, and capacity to integrate bioactive components. Oil-based hydrogels offer improved functionality for dental tissue regeneration and serve as scaffolds for GBR. In such circumstances, hydrogels function as scaffolds, providing a barrier that isolates the bone defect from soft tissue to facilitate optimal bone repair.

Overall, oil-based hydrogels demonstrated efficacy in the fabrication of 3D-printed scaffolds that facilitate bone regeneration, osteogenesis, and cellular proliferation. These scaffolds offer the essential structure and bioactive microenvironment for the restoration of bone defects, especially in the mandible, and several other dental applications. In various studies, hydrogels made from hydroxyapatite and other oils demonstrate mechanical stability comparable to that of natural bone, with some cases showing improved properties such as compressive strength, modulus, and shear yield strength. This renders them optimal candidates for structural reinforcement in bone tissue engineering. Some research highlights the use of antimicrobial oils (e.g., clove oil, tea tree oil, *Cannabis sativa* oil) into hydrogel compositions, enhancing their antioxidant and antibacterial characteristics. These advancements provide new possibilities for preventing infections in bone and dental restoration treatments.

### 4.2. Characterization of Oil-Based Hydrogels

The characterisation of oil-based hydrogels for bone and dental applications encompasses several characteristics, including viscosity, physical, chemical, thermal, and mechanical properties, which elucidate the material’s potential for 3D printing and biological applications. Oil-based hydrogels typically consist of plant-derived oils such as soybean or sunflower oil, acrylated epoxidized soybean oil (AESO), or synthetic oils. These oils provide hydrophobic properties that improve overall stability and facilitate emulsion formation. The amalgamation of oil-based matrices with biocompatible substances like hydroxyapatite and bioactive ceramics produces hybrid materials appropriate for bone and dental regeneration.

Hydrogels designed for bone and dental applications need substantial mechanical strength to endure the stresses applied during chewing or physical activity. Oil-based hydrogels demonstrate improved mechanical characteristics, including compressive strength and elasticity, through the formation of an interconnected, crosslinked structure with polymer matrices. Moreover, oil-based hydrogels exhibit advantageous rheological characteristics, facilitating their processing using several 3D bioprinting methods, including digital light processing (DLP) and extrusion-based 3D printing. These approaches allow the fabrication of bespoke scaffolds with exact designs that replicate the structure of real bone or dental tissues. The mechanical properties of the hydrogels exhibit considerable variability among various investigations. Li et al. (2023) observed that in AESO-based hydrogels, the mechanical strength, particularly compressive strength and modulus, decreased as the concentration of Ag-TMSPM-pBT nanoparticles increased [103]. The AESO-5ATP and AESO-10ATP scaffolds satisfied the mechanical criteria for bone restoration applications, exhibiting compressive strengths of 2.22 MPa and 1.38 MPa, respectively. The findings of Liu et al. (2023) further substantiated this, demonstrating that dHA scaffolds exhibited improved compressive strength and modulus, above the levels necessary for cancellous bone [104].

Oil-based hydrogels demonstrated non-Newtonian characteristics, with significant shear thinning evident in the samples. Neat soy oil had a low viscosity of 0.07 Pa·s at 1 s^−1^, which diminished further upon incorporation into other formulations. This feature is advantageous for 3D printing, as viscosity decreases under elevated shear rates, enabling extrusion via narrow nozzles while preserving its form after extrusion.

## 5. Strength and Limitations

A significant advantage of 3D bioprinting is its capacity to produce complex and individualized structures specific to patients. Bone and dental tissues possess complex microarchitectures that must be precisely replicated for effective integration and healing. 3D bioprinting can reproduce these intricate geometries, which is crucial for scaffolds or implants intended to conform precisely to faults or damaged regions. This customization enhances patient outcomes, especially in instances when conventional grafts or prosthesis may lack an accurate fit. It facilitates the creation of bio-scaffolds that align with the normal bone structure, enhancing tissue regeneration and integration. Moreover, oil-based hydrogels, derived from plant sources like soybean and sunflower oil, are crucial for medical applications due to their biocompatibility and ability to interact directly with body cells and tissues. They can also be biofunctionalized to enhance tissue regeneration, providing signals for cell growth, differentiation, and tissue repair.

A distinctive advantage of oil-based hydrogels is their frequent derivation from renewable, natural sources. In contrast to manufactured polymers, which can negatively affect the environment, the use of plant oils promotes the advancement of sustainable biomaterials. The increasing focus on eco-friendly and sustainable materials in biomedical engineering renders oil-based hydrogels an attractive choice. This attribute is advantageous not just for material manufacturing but also for advancing sustainable environmental objectives in eco-friendly healthcare solutions. Oil-based hydrogels exhibit significant versatility and may be tailored to possess certain mechanical properties that are appropriate for various applications. Modifying the hydrogel composition or integrating reinforcing agents like bioactive glass particles or cellulose nanocrystals enables these materials to replicate the mechanical properties of bone or tooth tissues. Certain oil-based hydrogels have been engineered to replicate the compressive strength and modulus of human bone, rendering them appropriate for bone healing. This tunability ensures that the hydrogels can deliver sufficient mechanical support in regions where stability is critical, such as load-bearing bones.

One advantage of oil-based hydrogels is their heat resilience, which is crucial for the sterilization processes. Numerous oil-based hydrogels are capable of enduring high temperatures, rendering them appropriate for autoclaving or other sterilizing techniques without jeopardizing their structural integrity. This feature is crucial for clinical applications, as any biomaterial designated for implantation must be sterile to prevent infections. The chemical stability of these materials enhances their endurance in the body, enabling the preservation of their structure and function over time.

Although oil-based hydrogels may be customized for their mechanical properties, they frequently do not match the performance of conventional materials utilized in bone grafting, particularly in load-bearing scenarios. The mechanical integrity of these hydrogels may be inadequate for areas subjected to significant stress, such as the long bones of the legs or substantial dental implants. Although reinforcing compounds can enhance the strength of these materials, they may still be deficient in the mechanical performance necessary for certain therapeutic applications, hence restricting their utilization to non-load-bearing areas or as temporary scaffolds.

Porosity is a crucial factor in tissue engineering, since it affects cellular infiltration, nutrition exchange, and waste elimination. Controlling the porosity of oil-based hydrogels presents significant challenges. Inadequate porosity may impede cellular migration into the scaffold, reducing the efficacy of tissue regeneration. Furthermore, degradation rates must be precisely regulated; excessive degradation might result in early scaffold failure, whereas insufficient degradation may hinder tissue development. Oil-based hydrogels may have inconsistent degradation rates, thereby affecting their efficacy in prolonged applications.

Attaining optimal printability is a significant problem in the 3D bioprinting of oil-based hydrogels. The viscosity and flow characteristics of these materials must be properly controlled to ensure smooth extrusion and layer deposition throughout the printing process. If the hydrogel exhibits excessive viscosity, it can block the printer nozzle; conversely, if it is overly fluid, the printed structure may lack stability and collapse. Maintaining the rheological characteristics of the hydrogel is crucial for preserving print quality and structural integrity; any alterations could affect the final scaffold’s shape and functioning.

## 6. Future Directions

Future advancements in 3D bioprinting of oil-based hydrogels for bone and dental applications should focus on many critical domains to improve the technology’s effectiveness and clinical relevance. The advancement of novel bioactive constituents for incorporation into oil-based hydrogels is essential. These components are intended to facilitate osteogenesis and dentinogenesis, hence enhancing integration with native bone and dental tissues. Secondly, enhancing the mechanical characteristics of oil-based hydrogels is crucial for ensuring that printed scaffolds can endure physiological loads and strains. This entails enhancing the hydrogel’s tensile strength, flexibility, and long-term stability.

Furthermore, enhancements in bioprinting methodologies and materials are essential to attaining greater accuracy and customisation in scaffold design, which has to be specifically adapted to individual patient requirements. The integration of growth factors, stem cells, and other regenerative agents into the hydrogel matrix may augment tissue regeneration and functional restoration. Ultimately, resolving regulatory and safety issues via thorough preclinical and clinical evaluations will be essential for the effective transition of 3D-bioprinted oil-based hydrogels from research to clinical use.

In addition, many previous studies provide a robust basis for further investigations aimed at enhancing oil-based hydrogels for clinical use. Subsequent research should prioritize expanding the range of in vivo models to encompass bigger animals, examining long-term impacts, and evaluating the efficacy of these materials under load-bearing situations pertinent to dental and bone applications. Moreover, the integration of advanced bioactive compounds and the functionalization of scaffolds with customized drug-release mechanisms could improve their therapeutic efficacy.

## 7. Conclusions

In summary, 3D bioprinting of oil-based hydrogels indicates an upcoming advancement in the development of complex scaffolding structures for bone and dental tissue engineering. The scoping review emphasizes the substantial advancements in enhancing the composition and printability of these hydrogels, along with their capacity to replicate the intricate microenvironment of bone and dental tissues. Nonetheless, additional obstacles persist, such as improving mechanical qualities, guaranteeing long-term stability, and including bioactive components to facilitate tissue regeneration. Subsequent study need be focused on overcoming these obstacles via novel material formulation, enhanced bioprinting methodologies, and comprehensive evaluation. Confronting these issues is essential for enhancing the clinical utilization of 3D-bioprinted oil-based hydrogels and eventually enhancing results in bone and dental tissue regeneration.

## Figures and Tables

**Table 1:** Summary of outcomes from oil-based hydrogel compositions utilizing various 3D bioprinting techniques.

No	Reference	Study Design	Type of 3D Bioprinting	Scaffold	Applications	Outcomes
1.	Ahmed et al. (2020) [105]	Biomaterial assessment	Extrusion-based 3D bioprinting	Bovine skin gelatin (BSG)/zinc oxide (ZnO)/clove essential oil (CEO) with alginate.	Tissue engineering	A gelatin-based antimicrobial film was adapted into 3D-printable ink for applications like food printing. This approach combines semi-solid extrusion with 3D printing, reducing development time and steps. The platform can be used with other drugs and biomaterials for personalized products.
2.	Mondal et al. (2021) [106]	Biomaterial assessment In vitro	Extrusion-based 3D bioprinting	Acrylated epoxidized soybean oil (AESO), Poly(ethylene glycol) diacrylate (PEGDA), and nano-hydroxyapatite (nHA)	Bone	The study developed 3D-printed nanocomposite scaffolds using AESO, nHA rods, and either HEA or PEGDA. The scaffolds showed good viscosity, particle dispersion, and mechanical strength. HEA improved shear yield strength, printability, cell adhesion, proliferation, and osteogenic differentiation, supporting bone tissue growth after 14 and 21 days.
3.	Antezana et al. (2022) [94]	Biomaterial assessment In vitro	Extrusion-based 3D bioprinting	Gelatin–Alginate Bioink with *Cannabis sativa* oil	NA	The study developed a gelatin–alginate bioink optimized for 3D bioprinting. The scaffolds can be lyophilized for storage without losing structure and have high absorption capacity for loading therapeutic molecules. Adding *Cannabis sativa* oil enhanced antioxidant and antimicrobial activity, making it a promising alternative to conventional treatments.
4.	Kim et al. (2022) [107]	In vitro In vivo	Extrusion-based 3D bioprinting (pneumatic pressure)	Porcine bone-derived dECM (BdECM) hydrgel-Ecs steroid in the mineral oi	Bone	Hybrid cell constructs with EC spheroids and hASC-laden dECM/β-TCP struts enhance bone formation and angiogenic activities, potentially serving as a therapeutic biomaterial for bone tissue induction.
5.	Kim and Kim (2022) [108]	Biomaterial assessment In vitro	Extrusion-based 3D bioprinting (pneumatic pressure)	Methacrylated collagen (CMA) bioink–mineral oil	Bone	The study developed a CMA-MO emulsion bioink for 3D cell constructs with human adenocarcinoma stem cells (hASCs). The bioink offers a stable structure with hierarchical pores, enhancing cell growth and cytoskeleton reorganization. It also delivers KGN and BMP-2, promoting chondrogenic and osteogenic differentiation, making it promising for improving cellular activities.
6.	Liu et al. (2023) [104]	Biomaterial assessment In vitro	Extrusion-based 3D bioprinting	(1) Hydroxyappatite (HA)-sunflower oil. (2) Hydroxyappatite (HA)-Pluronic^®^ F-127.	Bone	This study developed biomimetic hpHA scaffolds for bone tissue engineering using DIW. The scaffolds have interconnected macropores and micropores, with strength similar to cancellous bone. They enhance stem cell attachment, spreading, and growth, making DIW a promising method for BTE scaffold optimization.

**Table 2:** Summary of oil-based hydrogels: crosslinking mechanisms, rheological properties, as well as physical and chemical characteristics for 3D bioprinting in bone and dental applications.

No	Reference	Type of Crosslinker	Rheological Properties	Physical Properties	Chemical Characterization
1.	Ahmed et al. (2020) [105]	Cross-linked with 100 mM calcium chloride (CaCl_2_) solution	The print resolution of the printing materials was achieved to have layer height ~100 μm and the construct directly printed in a Petri dish containing 100 mM CaCl_2_ solution exhibited a good crosslinking (curing) behavior of the alginate at room temperature.	SEM: The addition of clove essential oil (CEO) into the bovine skin gelatin (BSG) matrix generated porosity, which could be related with the evaporation of the CEO during drying.	XRD: The neat gelatin film showed no distinct peaks, but zinc oxide (ZnO) and CEO composite films had characteristic peaks at 2θ of 10.4, 12.7, 22.8, 32.2, 34.8, 36.8, and 56.5. FTIR: All samples showed similar amide-band peaks (A, B, I, II, and III), with differences in wavenumber and peak intensity. The band at 1035 cm^−1^ indicated interactions between the film structure and glycerol’s O–H group vibrations.
2.	Mondal et al. (2021) [106]	Photocrosslinking	Adding 2-Hydroxyethyl Acrylate (HEA) and Polyethylene Glycol Diacrylate (PEGDA) reduced the viscosity of nanocomposite inks compared to pure Acrylated Epoxidized Soybean Oil (AESO)-based ones. HEA significantly lowered viscosity from 40.4 ± 0.88 Pa·s to 0.83 ± 0.24 Pa·s and increased shear yield stress from 11.33 ± 0.48 Pa to 68.33 ± 17.15 Pa.	SEM: The incorporation of HEA and PEGDA resulted in open-faced, well-defined, and interconnected porous networks.	FTIR: The spectra of the nanocomposites demonstrated successful curing as there were no peaks associated with vinyl groups remaining after curing.
3.	Antezana et al. (2022) [94]	Calcium chloride (CaCl_2_)	NA	SEM: The material had a compact, smooth surface with an average pore size of 15.0 ± 2.3 μm. Biodegradation: The gelatin– alginate (GEL–ALG) scaffold fully degraded in 5 h, while the gelatin–alginate–*Cannabis sativa* (GEL–ALG–CS) scaffold lasted up to 20 h, retaining 20% of its weight.	FTIR: Presence of characteristic peaks of CS at 2924 and 2850 cm^−1^, which is not observed in GEL-ALG.
4.	Kim et al. (2022) [107]	NA	NA	NA	NA
5.	Kim and Kim (2022) [108]	NA	The rheological properties abruptly decreased when the oil volume fraction in the emulsion bioink was above 30 *v*/*v*%.	SEM: Methacrylated collagen/mineral oil (CMA/MO) had a higher porosity (97.9 ± 0.3%) than CMA (96.4 ± 0.3%). Wettability: Dextran diffused faster in CMA/MO than in CMA, indicating better wettability. CMA/MO also showed higher protein absorption.	NA
6.	Liu et al. (2023) [104]	NA	All inks were shear-thinning. Hydroxyapatite (HA)-stabilized emulsions without Pluronic^®^ F-127 had higher viscosity, storage modulus, and yield stress after emulsification. More oil improved these properties but reduced HA content, limiting the viscosity increase.	SEM: 3D-printed HA scaffolds had pores of 300–400 μm horizontally and ~100 μm vertically. hpHA-P100 had more and larger micropores than F100, with higher oil content increasing micropores in the struts.	NA

**Table 3:** Summary of the temperature stability and mechanical properties of different oil-based hydrogel formulations that are used in 3D printing and tissue engineering.

No	Reference	Thermal Stability	Mechanical Characterization
1.	Ahmed et al. (2020) [105]	Bovine skin gelatin/zinc oxide/clove essential oil (BSG/ZnO/CEO) films showed three weight loss stages: 37.35% (197–256 °C), 31.51% (267–367 °C), and 18.41% (400–460 °C). ZnO reinforcement improved the thermal stability of the film.	Adding ZnO and CEO reduced the tensile strength of BSG films from 36.9 ± 2.8 to 32.7 ± 2.3 MPa (*p* < 0.05) but increased elongation at break from 15.05 ± 1.3% to 19.1 ± 1.8%.
2.	Mondal et al. (2021) [106]	NA	The representative stress-strain curves of S20 and SP20 nanocomposites demonstrate their higher tensile strengths compared to the SH20 nanocomposites. The tensile elastic moduli for S20, SH20, and SP20 were 689.95 ± 189.45 MPa, 198.72 ± 36.65 MPa, and 645.34 ± 149.78 MPa, respectively.
3.	Antezana et al. (2022) [94]	NA	NA
4.	Kim et al. (2022) [107]	NA	NA
5.	Kim and Kim (2022) [108]	NA	NA
6.	Liu et al. (2023) [104]	NA	dHA scaffolds had the highest compressive strength and Young’s modulus due to their dense struts and low porosity, exceeding those of cancellous bone (yield strength: 2–12 MPa, Young’s modulus: 50–500 MPa).

## Data Availability

Not applicable.
